# Gestational weight gain and its determinants among pregnant women attending antenatal care at West Shawa Hospitals, Oromia, Ethiopia

**DOI:** 10.1371/journal.pone.0323725

**Published:** 2025-06-09

**Authors:** Teka Girma Terfassa, Dessalegn Wirtu, Gudina Egata

**Affiliations:** 1 Department of Public Health, College of Health Sciences and References Hospital, Ambo University, Ambo, Ethiopia; 2 Department of Public Health Institute of Health, Wallaga University, Ethiopia; 3 Department of Nutrition and Dietetics, School of Public Health, College of Health Sciences, Addis Ababa University, Addis Ababa, Ethiopia; Ambo University, ETHIOPIA

## Abstract

**Background:**

Gestational Weight Gain (GWG) is a crucial factor influencing maternal and neonatal health outcomes. Identifying the determinants of GWG can help develop targeted interventions to improve pregnancy outcomes.

**Objective:**

This study aimed to assess the magnitude of gestational weight gain and identify its determinants among pregnant women attending antenatal care (ANC) services at West Shoa Hospital, Ethiopia, in 2024.

**Methodology:**

A bidirectional cohort study was conducted among 885 pregnant women attending antenatal care (ANC) services at West Shoa Hospitals, Ethiopia, before 12 weeks of gestation. Data were collected through face-to-face interviews using the CesPro application and document review. The determinants of GWG were analyzed using an ordinal logistic regression model, assuming the proportional odd assumptions. The Brant test was used to determine whether the parallel assumption was held. The STATA “ologit” command was used for ordinal regression, and the “brant” test was applied to verify the validity of the model. Odds ratios (OR) with 95% confidence intervals (CI) were estimated, and statistical significance was declared at p < 0.05.

**Results:**

Approximately 69% of pregnant women experienced insufficient weight gain, 26% had adequate weight gain, and 5% had excessive weight gain during pregnancy. Pre-pregnancy Body Mass Index was a significant determinant of gestational weight gain. Compared to underweight women, overweight women had 10.58 times higher odds (95% CI: 5.24–21.37) of being in a higher weight gain category, while obese women had 10.64 times higher odds (95% CI: 1.87–60.57) of achieving normal or excessive gestational weight gain. Partner education significantly influenced gestational weight gain, with those who could only read and write having 0.22 times lower odds (95% CI: 0.05–0.98) of excessive weight gain compared to those with higher education. Maternal occupation also played a role, as daily laborers had 0.26 times lower odds (95% CI: 0.08–0.87) of adequate weight gain than employed women. The normal hemoglobin category was associated with increased odds of being in a higher weight gain category (adequate or excessive) compared to a lower category, with an odds ratio (OR) of 1.04 (95% CI: 1.01–1.08). Conversely, alcohol consumption was associated with lower odds of being in a higher weight gain category, with an OR of 0.49 (95% CI: 0.25–0.99), suggesting that alcohol drinkers had lower odds of experiencing normal or excessive weight gain compared to non-drinkers.

**Conclusion:**

A significant proportion of pregnant women experienced inadequate gestational weight gain. Pre-pregnancy BMI, partner’s educational status, maternal occupation, hemoglobin levels, and alcohol consumption were key determinants of gestational weight gain. These findings highlight the need for targeted nutritional counseling and lifestyle interventions to promote optimal weight gain during pregnancy.

## Introduction

Gestational weight gain (GWG) is a critical factor affecting maternal and infant health, with variations in prevalence and patterns observed across different countries. In developed nations, excessive GWG is a growing concern, while inadequate weight gain remains a significant issue in many regions [1–3]. Scientific studies have demonstrated that insufficient GWG (gestational weight gain) can lead to short, mid, and long- term health implications for both mothers and o her offspring. Insufficient weight gain during pregnancy is associated with an increased risk of pregnancy-related complications and adverse birth outcomes, like low birth weight, preterm birth, and intrauterine growth retardation [4–7]. Excessive gestational weight gain can cause gestational hypertension, gestational diabetes, and complications of labor, delivery, and postpartum. Latin American country has a high burden of excessive gestational weight gain. In this region pregnancy-induced hypertension is common. About 66% of women gain excessive weight during their pregnancy time. Only 19.8% gained inadequate gestational weight [8].

In the developed world, “How much weight should I gain in this pregnancy?” is a question that many women ask their prenatal care providers today. The answer to this important question has changed dramatically over the past century. In the 1950s, one recommendation was for women to limit their weight gain to 10–14pound to avoid complications such as “toxemia” at a time when 10% of women with eclampsia died [9].

Gaining too much weight during pregnancy can produce immediate and long-term health risks to a woman and her infant [10]. An elevated pre-pregnancy BMI was reported to be linked to depression [11], preeclampsia, hypertension, gestational diabetes, and birth defects [12] while reports in the first trimester showed its association with outcomes, such as induction of labor, caesarean section [13] and higher odds of macrosomia [14]. Likely, findings from third-trimester pregnancy reveal that raised BMI was associated with higher chorioamnionitis and cesarean delivery rates, higher neonatal birth weight, and ponderal index [15]. Further, it was suggested to record both the weight and height of pregnant mothers as BMI may be predictive of gestational hypertension, preeclampsia, and gestational diabetes, large for gestational-age neonates at later trimesters. On the other hand, though inadequate weight gain poses little health risk to mothers, it may result in small growth-restricted infants, which increases the risk for infant mortality and developmental delay [16].

The International Institute of Medicine (IOM) established guidelines for gestational weight gain to enhance maternal and child health outcomes. In 2022, Ethiopia adopted these guidelines to address the high maternal and child mortality rates, which remain a critical public health challenge in the country. The guidelines provide specific recommendations for weight gain during pregnancy based on a woman’s pre-pregnancy body mass index (BMI). They aim to ensure that women gain an appropriate amount of weight during pregnancy, which can positively influence birth outcomes, reduce the risk of complications, and improve overall health for both mothers and their children. This is part of broader efforts to enhance healthcare quality and accessibility in the country [17,18].

The studies on gestational weight gain among pregnant women in Ethiopia reveal significant regional variations. In the Amhara region, about 65% of pregnant women gained adequate gestational weight, with an average increase of 13.3 kg. However, only 26% of pregnant women had inadequate weight gain compared to the Institute of Medicine (IOM) recommendations [19]. This finding contrasts sharply with studies from Addis Ababa and the Harari Regional State, where only about a quarter of pregnant women achieved adequate weight gain [20,21]. In the Guraghe Zone of Ethiopia, about half of the women gained normal gestational weight, further highlighting inconsistencies in findings across different regions of the country [22], maternal age, occupational status, and early pregnancy weight status were found to have a statistical significant association with the gestational weight gained.

The study addresses a significant gap in understanding the magnitude of gestational weight gain (GWG) during pregnancy, focusing on the amount of weight gained and the factors influencing this gain among pregnant women in the West Shoa, Oromia region. Despite the increasing recognition of the importance of GWG for maternal and child health, there is limited research on the magnitude and determinants of GWG in the West Shoa region of Oromia, Ethiopia.“ Moreover, the study aims to serve as a baseline for future research on the impact of gestational weight gain on pregnancy outcomes.

## Materials and methods

### Study area and setting

West Shoa is one of the zones of the Oromia Regional State. This zone is located 114 km from Finfinne/Addis Ababa, the capital city of Ethiopia, to the west. According to information from the West Shoa Health Office, this zone has a total population of **2.5** million, with around 1.2 million men and 1.3 women living within an area of 14,788.78 square kilometers. West Shoa has a population density of 139.21, while 342,352, or 13% are urban dwellers. Currently, there are about 495,753 women of reproductive age in this zone. There are around 100,283 mothers giving birth per year. It’s bounded via East, Shagar City, and South West Shoa, in the West; Horroo Guduruu and East Wollega Zones, in the northern part North shoa zone of the Oromia region.

The study area, in general, had 22 administrative districts and one town administration with over 528 rural kebeles and 58 urban kebeles found in it. In West Shoa Zone, there are nine public hospitals and 89 health centers currently providing health service. According to the West Shoa Zonal Health Department’s information, maternal health service utilization such as antenatal care visits, skilled birth attendances, and post-natal check-ups has shown to increase. This study was conducted from July 1, 2023, to March 30, 2024, among pregnant mothers of had ANC follow-ups.

### Study design

A bidirectional study design was employed to determine the magnitude of gestational weight among pregnant women attending ANC clinics in selected hospitals. A bidirectional cohort study is a special type that shares retrospective and prospective follow-up characteristics. Pre-pregnancy weight, date of last menstrual period, parity, and history of past obstetrics complications were assessed retrospectively, and the incidence of current pregnancy complications and adverse birth outcomes were assessed through follow-up. As a hybrid model, this type of cohort study has advantages in that lifestyle habit studies can be categorized as ambispective cohort studies since the exposure—which would in most cases, continue to develop over time—has already occurred before the study baseline [26].

#### Source population.

All pregnant women who attended antenatal care follow-up in the West Shoa hospitals during the study period were our source population for the study.

#### Study population.

All selected pregnant women who attended antenatal care follow-up in the selected West Shoa hospitals during the study period and those who fulfilled the inclusion criteria.

### Inclusion and exclusion criteria

#### Inclusion criteria.

The study included women who were on ANC and with singleton pregnancies who were residents of the study area and planned to remain there until delivery. Additionally, only those who initiated antenatal care (ANC) follow-up before 12 weeks of gestation were eligible for inclusion.

#### Exclusion criteria.

Women with pre-existing or current medical conditions such as hypertension (HTN) and diabetes mellitus (DM), as well as those twin pregnancies, were excluded from the study based on ultrasound findings. Those second visits and above were excluded from this research.

### Sample size determination

The sample size was calculated using the two-population proportion formula, based on the following assumptions: a 12.4% prevalence of preeclampsia among unexposed individuals and a 6.4% prevalence among exposed individuals, as reported in a systematic review of sab-Saharan Africa by Fekede A. et al. [23]. The study employed a 1.1 ratio of exposed to non-exposed individuals. After adjusting for a 10% non-response rate, the final sample size was set at 885.

### Sampling technique

The pregnant women were selected from 4 out of 9 hospitals found in West Shoa. The facilities were selected based on their ANC caseload and the level of hospitals (Primary Hospital, General Hospital, and Referral Hospitals) in the study area. Women who met the inclusion criteria were consecutively selected from each health facility until the required sample size was met. All pregnant women were invited during their first trimester (before 12 weeks gestation) among ANC visitors at the selected health facilities. The staff of the health facilities in charge of providing antenatal care participated in the facilitated study participant selection process.

#### Dependent variables.

- Gestational weight gain status

#### Independent variables.

- **Socio-demographic factors** (age, height, weight, residence, religion, educational level, marital status, educational level of the partner occupation, occupation of the partner, monthly income, living with partner, dietary diversity, food security, perinatal depression
- **Obstetrics and medical-related factors (**Parity, multiple gestation, gestational diabetes mellitus (GDM), history of complication in previous pregnancy, birth interval, history of abortion, history of stillbirth.

### Data collection procedure

The data was collected through face-to-face interviews using a pre-prepared CesPro application tool developed by trained professionals. Additionally, relevant variables were obtained by reviewing health records, and anthropometric measurements, such as height, weight, and MUAC (Mid-Upper Arm Circumference), were taken. Height was measured using a stadiometer with participants standing barefoot, ensuring the head was positioned in the Frankfurt plane. Measurements were recorded to the nearest 0.1 cm. Weight was measured using a calibrated digital weighing scale, with participants wearing light clothing and no shoes. Measurements were recorded to the nearest 0.1 kg. MUAC was measured using a non-stretchable measuring tape placed around the midpoint between the acromion process and the olecranon of the left upper arm. The measurement was recorded to the nearest 0.1 cm.

The CesPro app is a mobile application designed to streamline and improve the process of documenting and managing delivery and other obstetric procedures in healthcare settings. It is typically used by healthcare professionals, including obstetricians, gynecologists, and midwives, to collect data on C-sections, monitor patient outcomes, and ensure the quality of care provided during and after surgical deliveries.

The app can include features such as:

- Patient Record Management: Securely storing patient data, including medical history, surgery details, and follow-up care.

### Data collection tools

A questionnaire was initially prepared in English after reviewing the literature and then translated into the Afan Oromo, the regional language, by a language expert. To ensure consistency, a back translation was conducted. The questionnaire comprises various sections, including socio-demographic characteristics, reproductive health, dietary diversity and food security, intimate partner violence, physical activity, and depression-related symptoms.

Variables such as gestational age (ultrasound result), blood pressure, random blood sugar, anemia status, and HIV status were obtained from the medical records of the women. The principal component analysis was employed to compute a wealth index from a set of household assets questions such as electricity, refrigerator, table, chair, watch, phone, bed with mattress, electric bakery (an Ethiopian oven made up of clay and metal), car, house, improved water, and improved toilet, which was be adapted from the Ethiopian demographic and health survey [24].

### Data quality assurance

Data collectors with previous experience in data collection were recruited for all data collection components. Training was provided to data collectors and supervisors on the study overview and details about tools and measurements. The tool was pre-tested on 5% of the calculated sample size in institutions not selected for the actual study before data collection. Supervisors checked the data for completeness and guided data collectors in case of difficulties.

Finally, the principal investigator monitored the overall quality of the data. For the data extraction component, data were collected by nurses and midwives with ample experience in clinical practice and data collection. Intensive training was given to data collectors and supervisors on how to extract data from medical records. Continuous supervision was conducted during the data collection period. The supervisors ensured data completeness and consistency daily.

#### Data management and analysis.

Data was exported from the database into SPSS version 24 for data cleaning and STATA 14.0 were used for analysis. Descriptive statistics, including figures, frequencies, and percentages, were calculated and the results were presented using tables and figures. An ordinal logistic regression analysis model was used to identify determinants of gestational weight gain. The odd ratio was calculated, and a P-value of less than 0.05 was considered statistically significant. Multicollinearity was assessed using the variance inflation factor (VIF) which was found to be less than 1.014, indicating no multicollinearity among variables. Model fitness was evaluated using the Pearson and Deviance goodness-of-fit tests; the model fitting information was found to be a significant p-value of 0.001). The parallel lines assumption, a key assumption in ordinal logistic regression, was tested using the Brant test to ensure that the proportional odds assumption was met. A non-significant p-value from the Brant test confirmed that the assumption was satisfied, allowing for the interpretation of a single set of odds ratios across weight gain categories.

#### Operational definitions and measurements.

**Gestational weight gain (GWG**) was ascertained using the International Institute of Medicine criteria. Based on pre-pregnancy body mass index (BMI) classes.

- Insufficient weight gain
- Adequate weight
- Excessive weight gain [25,26]

**Adverse perinatal outcome** was defined with a composite measure based on the presence of one or more of the following: stillbirth, low birth weight, preterm birth, admission to neonatal ICU, and first-minute birth asphyxia.

Body Mass Index (BMI) is the ratio of weight in kg to height in a meter square [25].

**Stillbirth** was defined as a newborn with no signs of life at or after 28 completed weeks of pregnancy.

**Low birth weight** was defined as a newborn’s weight below 2500 grams.

**Preterm** birth was defined as a baby born alive before 37 weeks of gestation but after 28 weeks of gestation.

**Gestational age** was determined based on the last menstrual period, with ultrasound measures used when prediction by the last menstrual period was not possible.

**Perinatal asphyxia** severity in newborns was graded using the Apgar score. A score below 7 at the first minute of life was considered as having first-minute birth asphyxia.

### Ethics approval and consent to participate

Ethical approval for the study was granted by the Wallaga University Institutional Health Research Ethics Review Committee (WU/RD/675/2023). In alignment with ethical standards, all participants provided written informed consent, ensuring voluntary participation. The study was conducted in a private setting to protect participants’ confidentiality, following permissions obtained from all relevant health facilities managers.

## Results

### Socio-demographic characteristics of the study participants

In this study, 885 pregnant women were interviewed, with a response rate of 100%, meaning all participants who were approached for the study provided the necessary data at the start of the follow-up of the study as a baseline. The participants’ ages ranged from 16 to 45 years, with a mean age of 25.4 years (± 4.55 SD). More than half 454, 51.1%) of the participants were between 19 and 25. The majority, 684 participants (85.1%), identified as Oromo by ethnicity, and 525 (65.3%) were Protestant Christian. About one-third, 276 (30.8%) of the pregnant women had attained a college education or higher. The number of pregnant women followed until the end was 797 from 885. The distribution of pregnant women by age, marital status, gravidity, and residence is presented in the table below (Table 1).

**Table 1:** Socio-demographic characteristics of gestational weight gain among pregnant women following ANC in West Shoa public hospitals, Oromia, May 2024 (n = 885).

Variable	Categories	Frequency	Percentage
Age	15-18	45	5.1
	19-25	454	51.3
	26-35	359	40.6
	36-45	27	3.1
Ethnicity (885)	Oromo	754	85.2
	Amhara	105	11.9
	Tigree	12	1.4
	Gurage	13	1.5
	Others *	1	0.1
Religion (885)	Orthodox	261	29.5
	Protestant	581	65.6
	Muslim	29	3.3
	Catholic	5	0.6
	Waqefata	9	1
Marital status (885)	Never married	18	2
	Married	865	97.7
	Divorced/separated	2	0.3
Educational status	Unable to read and write	62	7
	Able to read and write	63	7.1
	Primary education (1–8)	279	31.5
	Secondary education	205	23.2
	College and above	276	31.2
Partner educational status	Unable to read and write	43	4.9
	Able to read and write	34	3.8
	Primary Education(1–8)	213	24.1
	Secondary education	204	23.1
	College and above	391	44.2
Maternal occupation	House wife	399	45.1
	Gov’t/private employer	273	30.8
	Merchant	137	15.5
	Student	29	3.3
	Daily labourer	37	4.2
	Others ***	10	1.1
Residence	Urban	659	82.0
	Rural	143	17.8
Wealth status(PCA)	Poor	259	32.2
	Medium	276	34.3
	Rich	269	33.5

### Maternal characteristics and weight gain

Most of the respondents (65.7%) have experienced 0–2 pregnancies prior to this pregnancy. About 250 (31.1%) of the pregnant women have experienced 3–5 pregnancies before this pregnancy. Slightly more than half of the respondents (51.0%) had pregnancy intervals of less than 23 months. The vast majority (96.0%) did not have a history of low birth weight (LBW). A minority of respondents (11.8%) reported having a history of abortion. A small percentage (4.7%) of respondents reported having a history of stillbirth. The mean pre-pregnancy weight of the study participants was 55.81 kg with a standard deviation of ±0.28 kg. The mean weight at the last recorded measurement of the study participants was 65.43 kg with a standard deviation of ±0.34 kg.

Overall, the data highlights that most respondents have had a relatively low number of pregnancies, with approximately equal distribution between shorter and longer pregnancy intervals. Histories of adverse pregnancy outcomes such as LBW, abortion, and stillbirth are relatively less among the respondents (Table 2).

**Table 2:** Maternal characteristics of study participants attending ANC follow-up Public Hospitals in West Shoa, Oromia, Ethiopia, 2024.

Variable	Category	Frequency	Percentage
Gravidity	0-2	528	65.7
	3-5	250	31.1
	>5	26	3.2
Pregnancy interval	>23 months	410	51.0
	≥24moths	394	49.0
History of having LBW	Yes	32	4
	No	771	96
History of abortion	Yes	95	11.8
	No	709	88.2
History of still birth	Yes	38	4.7
	No	766	95.3

### History of maternal depression during pregnancy

The data provides the prevalence and severity of maternal depression during pregnancy among 885 respondents. The respondents are categorized based on the severity of depression experienced: no depression, moderate depression, and severe depression. A total of 290 (36.1%) of the respondents, reported experiencing no depression during their pregnancy, while 360 (44.8%) experienced moderate depression during pregnancy.

Severe depression was reported by 154 (19.2%) of the respondents. Severe depression during pregnancy can have serious implications for both the mother and the developing fetus, including risks of poor prenatal care, preterm birth, and low birth weight.

**Fig 1:**
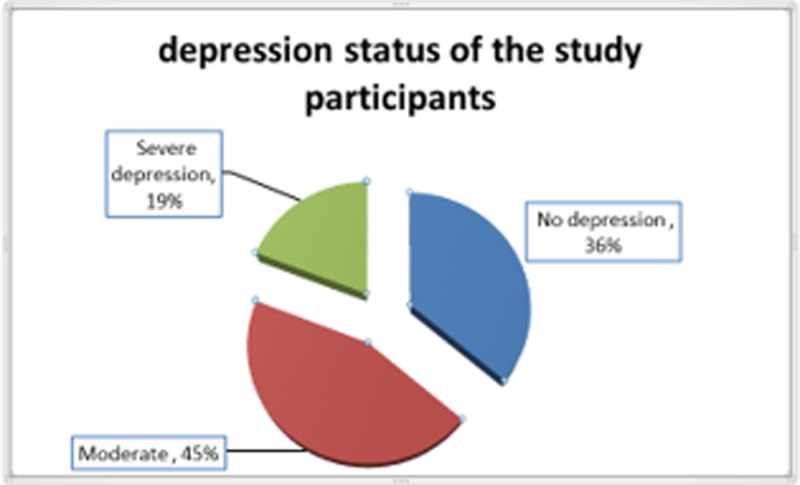
Depression status of pregnant women who were on ANC follow-up in the public Hospital of West Shoa, Oromia, Ethiopia, 2024.

With nearly 398(45%) experiencing moderate depression and about 154 (19%) facing severe depression, there is a clear need for targeted mental health interventions. These could include screening for depression as a routine part of prenatal care, providing counselling services, support groups, and when necessary, medical treatment to manage symptoms and improve outcomes for both mothers and their babies (Fig 1).

### Women’s dietary diversity (DD) assessment

The mean women’s dietary diversity score of study participants was 5.03 (± 1.4SD). Among the participants, 448 (55.7%) and 356 (44.3%) had adequate and inadequate dietary diversity scores, respectively. Regarding the consumed food groups by pregnant women in the previous 24 hours, nearly all women, 720 (89.9%), consumed starchy staples, 733 (91.2%) of women consumed pulses like peas and beans as sauce for their meal, 718 (89.3%) consumed Any foods made with oil or butter. Low feeding practice was observed in any fish and animal sources like meat and beef foods in 18% and 45%, respectively (Table 3).

**Table 3:** Dietary diversity assessment of pregnant women who were on ANC follow-up in the public hospital of West Shoa, Oromia, Ethiopia, 2024.

Types of food consumed in 24 hours	Response category	Frequency	Percentage
Ate starchy staples	No	94	10.6
	Yes	791	89.4
Ate any potatoes/roots or tubers	No	112	12.7
	Yes	773	87.3
Ate Any vegetables	No	223	25.2
	Yes	605	74.8
Ate Any fruits	No	290	32.8
	Yes	595	67.2
Ate any meat/beef	No	480	54.2
	Yes	405	45.8
Ate egg/s	No	293	33.1
	Yes	592	66.9
Ate fish	No	726	82.0
	Yes	159	18.0
Ate Any foods made from beans, peas	No	71	8.8
	Yes	733	91.2
Ate any milk and milk product	No	297	33.6
	Yes	588	66.4
Ate any foods made with oil or butter	No	94	10.6
	Yes	791	89.4
Ate Any sugar or honey	No	335	37.9
	Yes	550	62.1
Ate Any other foods condiments, coffee, tea	No	65	7.3
	Yes	820	92.7

### Household food insecurity access

The study revealed significant levels of food insecurity among pregnant women, with 10% worrying about not having enough food and 12.3% unable to obtain their preferred foods in the past 30 days. Additionally, 13.6% had to eat a limited variety of foods, 7.7% consumed foods they did not want to eat, and 8.6% ate smaller meals than necessary. Furthermore, 8.7% ate fewer meals daily, 4.4% experienced having no food at all, 3.8% went to bed hungry, and 4.7% went a whole day and night without eating (Table 4).

**Table 4:** Household food insecurity access of pregnant women who were on ANC follow-up in the public hospital of West Shoa, Oromia, Ethiopia, 2024.

Types of food	Response category	Frequency	Percentage
Worry not having enough food	Yes	88	9.9
	No	797	90.1
Not able to eat preferred foods	Yes	108	12.2
	No	777	87.8
Eat a limited variety of foods	Yes	122	13.8
	No	763	86.2
Eat some foods not want to eat	Yes	68	7.7
	No	817	92.3
Eat smaller meals than felt	Yes	75	8.5
	No	810	91.5
Eat fewer meals in a day	Yes	75	8.5
	No	810	91.5
No food to eat any kind	Yes	39	4.4
	No	846	95.6
Sleep at night hungry	Yes	34	3.8
	No	851	96.2
whole day & night without eating anything	Yes	41	4.6
	No	844	95.4

### Weight gain status of the study participants

**Fig 2:**
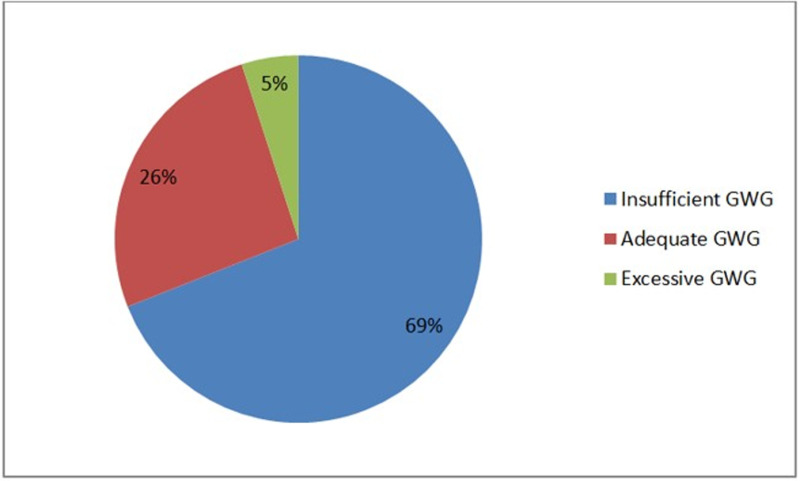
Percentage of gestational weight gain status of pregnant women who were on ANC follow-up in the public hospital of West Shoa, Oromia, Ethiopia, 2024.

In the present study, the level of gestational weight gain among pregnant women was categorized into three groups: about 69% of the women were insufficient, meaning they gained less weight than recommended during pregnancy, 26% had an adequate weight gain, meeting the recommended levels, 5% of the women overweight gained which means more than the recommended weight during pregnancy (Fig 2).

### Weight gain compared with pre-pregnancy BMI

The key findings indicate that a significant portion of the study population did not achieve the recommended gestational weight gain. Among participants with a normal pre-pregnancy BMI, the majority (395 out of 536) experienced insufficient weight gain, a trend also observed among those with a low pre-pregnancy BMI (133 out of 183). Conversely, participants who were overweight or obese before pregnancy were more likely to gain excess weight during gestation, with 21 out of 66 overweight and 6 out of 11 obese participants exceeding the recommended weight gain. These results suggest that both underweight and overweight women are at risk of not meeting optimal gestational weight gain, emphasizing the need for tailored nutritional and health interventions during pregnancy (Table 5).

**Table 5:** Comparison of pre-pregnancy BMI with gestational weight gain of pregnant women on ANC follow up in West Shoa zone public hospitals, Oromia, Ethiopia 2024.

Pre-pregnancy weight BMI compare with gestational weight gain					
		Weight gain category			Total
		Insufficient weight gain	Normal weight gain	Overweight gain	
Pre-pregnancy category	Low weight	133	42	8	183
	Normal	395	135	6	536
	Over weight	18	28	21	66
	Obesity	5	0	6	11
Total	551	205	41	797

### Relationship between weight gain and different categories of variables

The study examined gestational weight gain (GWG) among pregnant women, categorizing weight gain as insufficient, adequate, or excess, and assessed its correlation with various socio-demographic and health-related variables, such as marital status, educational status, partner’s educational status, occupation, residence, pregnancy interval, HIV status, prenatal depression status, dietary diversity, and pre-pregnancy weight status. For instance, among never-married women, 64.7% experienced insufficient weight gain, 29.4% had adequate weight gain, and 5.9% had excess weight gain. In contrast, women whose partners had a college education or higher showed 70.6% insufficient weight gain, 23% adequate weight gain, and 6.5% excess weight gain. These findings highlight significant variations in weight gain patterns, emphasizing the impact of educational status, occupation, and other factors on maternal health during pregnancy (Table 6).

**Table 6:** Proportion of weight categories to different variables among pregnant women on ANC follow up in West Show Hospital, 2024.

Variables		Proportions of GWG		
		Insufficient WG (%)	Adequate WG (%)	Excess WG (%)
**Marital status**	Never married	11 (64.7)	5 (29.4)	1 (5.9)
	Married	490 (69.6)	177 (25.1)	37 (5.3)
	Divorced/separated	2 (100)	0 (0)	(0)
**Maternal educational status**	Unable to read and write	31 (54.4)	21 (36.8)	5 (8.8)
	Able to read and write	42 (82.4)	9 (17.6)	0 (0)
	Primary education (1–8)	175 (72.9)	53 (22.1)	12 (5)
	Secondary education	113 (67.3)	54 (32.1)	1 (0.6)
	College and above	142 (68.6)	45 (21.7)	20 (9.7)
**Partner educational status**	Unable to read and write	15 (41.7)	17 (47.2)	4 (11.1)
	Able to read and write	25 (86.2)	4 (13.8)	0 (0)
	Primary education(1–8)	135 (73)	41 (22.2)	9 (4.9)
	Secondary education	110 (67.1)	49 (29.9)	5 (3)
	College and above	218 (70.6)	71 (23)	20 (6.5)
**Maternal occupation**	Housewife	221 (66.8)	94 (28.4)	16 (4.8)
	Government/Private employee	152 (71.7)	49 (23.1)	11 (5.2)
	Merchant	81 (71.1)	26 (22.8)	7 (6.1)
	Student	14 (56)	7 (28)	4 (16)
	Daily labourer	28 (87.5)	4 (12.5)	0 (0)
	Others	7 (77.8)	2 (22.2)	0 (0)
**Residence**	Urban	394 (67.5)	156 (26.7)	34 (5.8)
	Rural	109 (78.4)	26 (18.7)	4 (2.9)
**Pregnancy interval**	0-23 months	272 (71)	87 (22.7)	24 (6.3)
	24months and above	231 (67.9)	95 (27.9)	14 (4.1)
**HIV status**	Reactive	5 (71.4)	2 (28.6)	0 (0)
	Nonreactive	466 (70.6)	160 (24.2)	34 (5.2)
	Not determine	32 (57.1)	20 (35.7)	4 (7.1)
**Prenatal Depression status**	No depression	179 (67.5)	69 (26)	17 (6.4)
	Mild	226 (69.8)	84 (25.9)	14 (4.3)
	Severe	98 (73.1)	29 (21.6)	7 (5.2)
**Dietary diversity status**	Poor dietary diversity	220 (71)	72 (23.2)	18 (5.8)
	Good dietary diversity	283 (68.5)	110 (26.6)	20 (4.8)
**Pre pregnancy BMI**	Low weight	133 (73.5)	42 (21.7)	8 (4.8)
	Normal weight	395 (74)	135 (24.8)	6 (1.2)
	Over weight	18 (26.7)	28 (41.7)	21 (31.7)
	Obesity	5 (44.4)	0 (0)	6 (55.6)

### Factors associated with gestational weight gains

The ordinal logistic regression analysis was conducted to identify factors significantly associated with weight gain categories. Pre-pregnancy BMI is a critical determinant, with overweight and obesity showing strong positive associations. Being overweight increases the odds of normal weight gain approximately 10.58 times (95% CI: 5.24, 21.37), P-value:0.001, and being obese further increases this risk to about 10.64 times (95% CI: 1.87, 60.57) P-value:0.008). Pre-pregnancy BMI was a significant determinant of gestational weight gain (GWG). The ordinal logistic regression model indicated that higher pre-pregnancy BMI was positively associated with being in a higher GWG category (adequate or excessive) compared to a lower category (insufficient).

Compared to underweight prepregnant women, overweight BMI women had 10.58 times higher odds (OR = 10.58, 95% CI: 5.24–21.37, p = 0.001) of being in a higher weight gain category. Similarly, obese women had 10.64 times higher odds (OR = 10.64, 95% CI: 1.87–60.57, p = 0.008) of experiencing normal or excessive GWG compared to underweight women.

Women whose partners had only basic literacy (able to read and write) had 78% lower odds (OR = 0.22, 95% CI: 0.05–0.98, p = 0.0045) of being in a higher weight gain category (normal or excessive) compared to those whose partners had a university degree or higher. Maternal occupation was significantly associated with gestational weight gain. Compared to employed women, daily laborers had 74% lower odds (OR = 0.26, 95% CI: 0.08–0.87, p = 0.026) of being in a higher weight gain category (adequate or excessive).

Additionally, normal hemoglobin levels showed a positive association with GWG, women with normal hemoglobin category had 4% higher odds of being in a higher gestational weight gain (GWG) category (adequate or excessive) compared to those with low hemoglobin levels (OR = 1.04, 95% CI: 1.01–1.08, p = 0.022).

A history of alcohol consumption is also significantly associated with reduced weight gain, reflected by 0.49% times decrease in odds of achieving normal or excessive weight gain (95% CI: 0.25, 0.99), P-value: 0.043. These findings highlight the multifaceted nature of weight gain during pregnancy, influenced by pre-pregnancy BMI, religious affiliation, partner’s education, maternal occupation, hemoglobin levels, and alcohol consumption history (Table 7).

**Table 7:** Ordinal Logistic regression analysis of gestational weight gain status for pregnant women who were on ANC follow up in the public hospital of West Shoa, Oromia, Ethiopia, 2024.

Variables	AOR	Standard Error	P value	[95%Conf. Interval]
Pre pregnancy BMI				
**Normal**	0.863	0.195	0.515	(0.554, 1.344)
**Overweight**	10.732	3.858	0.000	**(5.304, 21.710)**
**Obesity**	10.633	9.419	0.008	(1.873, 60.348)
**Minimum Dietary diversity**	1.209	0.229	0.315	(0.834, 1.753)
Wealth index				
**Medium**	0.888	0.195	0.588	(0.576, 1.365)
**Rich**	1.111	0.245	0.632	(0.721, 1.711)
**Marital_status**				
**Married**	0.753	0.457	0.641	(0.229, 2.475)
**Divorced/separated**	1.93	0.004	0.994	0
Partner educational status				
**Able to read and write**	0.211	0.163	0.045	**(0.046, 0.963**)
**Primary education (1–8)**	0.470	0.278	0.203	(0.148, 1.497)
**Secondary education**	0.621	0.376	0.432	(0.189, 2.035)
**College and above**	0.534	0.325	0.304	(0.162, 1.763)
**Maternal_occupation**				
**Employee**	0.662	0.166	0.102	(0.403, 1.085)
**Merchant**	0.802	0.219	0.423	(0.469, 1.373)
**Students**	1.851	0.836	0.173	(0.763, 4.488)
**Daily labourer**	0.255	0.156	0.026	**(0.08, 0.85)**
**Others**	0.490	0.472	0.46	(0.07, 3.24)
**Rural residence**	0.699	0.188	0.185	(0.41, 1.19)
**History of abortion**	0.950	0.095	0.618	0.78, 1.16
**Vigorous activities**	1.105	0.084	0.187	0.95, 1.28
Having depression				
**Mild**	0.944	0.185	0.773	0.64, 1.39
**Severe**	0.756	0.198	0.287	0.45, 1.26
**Positive for HIV**	1.605	0.514	0.14	0.86, 3.01
**Having anaemia**	1.042	0.019	0.022	**1.01, 1.08**
**Hist. alcohol**	0.489	0.172	0.043	**0.245, 0.98**
**History of cigarette smoking**	2.459	3.070	0.471	0.213, 28.43
**MUAC less than 23 cm**	1.056	0.210	0.784	0.715, 1.56
**/cut1**	1.583	2.382		−3.09, 6.25
**/cut2**	4.095	2.388		−0.586, 8.78

## Discussion

A study was conducted to assess gestational weight gain status in West Shoa Zone public hospital from July 2023- March 2024 among pregnant mothers of had ANC follow-up. 885 participants were initially interviewed, achieving a 100% response rate. Of these, 797 participants (90.1%) were followed until the outcome. The age of the participants ranges from 16–45 years with a mean age of 25.4 years (+ 4.55SD). The majority of the study participants 97.7% and 82% were married and urban dwellers respectively.

In our study, inadequate gestational weight gain was prevalent, with over two-thirds of women experiencing insufficient weight gain during pregnancy, about 205(26%) had adequate weight gain, and 41(5%) gained more weight than recommended. This finding closely align with the study conducted at Addis Ababa where 67.2% gained inadequate weight, 27.9% gained adequate weight and 4.9% of the pregnant women gained excessive weight [20]. Similarly, a study conducted in the Tigray region reported that 64% of pregnant women had insufficient weight gain. In contrast, studies in North Ethiopia, South Godar zone, and Guraghe zone showed a higher percentage of women gaining adequate weight (56%) gained [22] and Additionally, findings from Colorado state showed higher percentage of adequate gestational weight gain than our study [27]. The reason might be due to economic instability since COVID-19 in the area, food insecurity, rural-to-urban internal displacement of the community for the sake of war between the government army and rebellion front in the area, and sociocultural factors. Therefore, addressing these issues through targeted interventions is crucial to improve maternal and fetal health outcomes.

The present study suggests that the majority (69%) of the study population did not achieve the recommended weight gain or had insufficient weight gain. Weight gain was stratified based on the pre-pregnancy BMI of the study population, about 73% of the low BMI gained insufficient weight similar finding was observed in Osun state of Nigeria where about 96.6% of the study population gained less than the recommended weight from the low pre-pregnancy [28] and a systematic review conducted in sub-Saharan Africa also suggests that among underweight pregnant women show that inadequate gestational weight gain varied between 67% to 98% [23]. This may be due to pregnant women with low pre-pregnancy weight having existing nutritional deficiencies or inadequate nutrient stores, which can make it difficult to gain the necessary weight during pregnancy also low body weight often, have higher metabolic rates, meaning they may burn calories faster, making it harder to accumulate the necessary weight.

The ordinal logistic regression analysis identifies several significant factors influencing weight gain categories among pregnant women. Key determinants include pre-pregnancy BMI, religious affiliation, partner’s educational status, maternal occupation, hemoglobin levels, and alcohol consumption history.

The percentage of women who gained the IOM-recommended amount of weight was highest among those with a normal BMI (65.8%) and lowest among those in the obese group. This finding is consistent with the study conducted in Ankara, Turkey [29] and the study conducted in Addis Ababa, Ethiopia where being underweight or normal weight before pregnancy increased the odds of gaining inadequate gestational weight compared to overweight or obese women [20]. The pre-pregnancy weight status is highly connected to maternal nutrition before conceptions which have an impact later on the amount of weight gain. According to the IOM guidelines, women who were underweight before pregnancy are expected to gain more weight to reach the normal category compared to those who were overweight or obese. In contrast, overweight and obese women have lower recommended weight gain targets, making it relatively easier for them to achieve the expected range of healthy weight gain. This study highlights the significant impact of pre-pregnancy BMI on gestational weight gain. Overweight and obese women before pregnancy were at higher odds of experiencing increased weight gain categories, emphasizing the need for BMI management before pregnancy to prevent unhealth weight gain pattern. Effort is needed to develop a strategy and promote adequate weight gain for underweighted pregnant women is needed to Ethiopia in general. Women whose partners have limited education (reading and writing only) exhibit reduced odds of normal weight gain categories when compared to their counterparts. Having an educational background has increased nutrition awareness and dietary diversity [30]. A study conducted in northern California [31] and a systematic review and meta-analysis conducted recently show that there is a strong association between educational status and gestational weight gain [32]. This might be due to educational levels are often correlated with better economic status, providing access to healthier food options, prenatal care, and health services. Education can lead to better lifestyle choices, such as regular physical activity, a balanced diet, and avoidance of harmful substances, all of which contribute to healthy gestational weight gain.

Occupations involving daily labor show a significant negative association with normal weight gain. Daily laborers are more likely to decrease the chance of normal weight gain or over weight gain category. The finding is consistent with study conducted in China, show that those who were unemployed, house work or temporary workers have increased odds of inadequate gestational weight gain when compared to their counter parts [33] and prospective study conducted in Addis Ababa [20], study conducted in Rio de Janeiro [34]. This may be to higher physical activity levels or job-related stress affecting nutritional intake and metabolic processes during pregnancy. Providing good job opportunities for women can increase the chance of gaining appropriate weight during pregnancy.

Higher hemoglobin levels are associated with slightly increased odds of normal or overweight gain categories when compared to those having low hemoglobin levels. The finding is consistent with a cohort study conducted in China that reported that a higher rate of GWG was associated with higher hemoglobin [35] but a study conducted in a state in Brazil reported that there is not observed significant association between them [36]. This finding suggests that maternal health conditions related to iron levels or nutritional status may play a role in shaping weight gain patterns during gestation.

Gestational weight gain for women with a history of alcohol consumption exhibit reduced odds of normal or overweight gain categories, i.e., more likely to have inadequate weight gain when compared to those not having a history of alcohol consumption. This finding is consistent with the study conducted in South Gondar [19] and alcohol consumption may lead to another behavioral problem that can pose nutritional problems [37]. Alcohol consumption has significantly positively predicted depressive symptoms which lead to poor appetite and hopelessness [38]. This underscores the potential impact of alcohol consumption patterns on metabolic processes or dietary choices influencing weight gain during pregnancy.

This study has the advantage of using a large sample size and prospective cohort study design. However, the major limitation that should be noted is that several of the critical items in the data set are self-reported, most significantly pre-pregnancy weight and weight gain in pregnancy. Ideally, gestational weight gain should be determined by comparing a woman’s weight just before delivery with her weight at conception. In our research setting, where preconception care is uncommon, we used the early pregnancy weight (before 12 weeks gestation) as pre-pregnancy weight. While this method might result in an underestimation of actual gestational weight gain, the first 12 weeks of pregnancy typically involve minimal weight gain, so this limitation is unlikely to significantly affect the validity of our results. Due to the few numbers of pregnant women receiving early antenatal care, we used consecutive sampling of those who met the inclusion criteria to ensure an adequate sample size. Since the timing of antenatal care visits is generally random, this approach should not impact the validity of our findings.

## Conclusions

Approximately 69% of pregnant women experienced insufficient weight gain, 26% had adequate weight gain, and 5% had excessive weight gain during pregnancy. The majority of pregnant women are not gaining enough weight for optimal pregnancy outcomes, suggesting the need for improved awareness and support regarding healthy weight gain during pregnancy. Monitoring and addressing weight gain can help reduce the risk of complications for both mother and baby. Factors significantly associated with gestational weight gain included pre-pregnancy BMI (overweight and obesity), partner’s educational status, daily labor, hemoglobin level, and alcohol consumption.

To promote healthy gestational weight gain, all women of reproductive age should be educated on maintaining a normal BMI before pregnancy. Encouraging partner education, reducing strenuous physical activities like daily labor, and promoting balanced nutrition to support optimal hemoglobin levels are essential. Health professionals should adhere to Ethiopia’s antenatal care guidelines to prevent unintended gestational weight gain and ensure better maternal and fetal health.

### Recommendations

Based on these findings, several recommendations can be made to promote healthy weight management during pregnancy:

Provide targeted counseling on body mass index (BMI) management and healthy lifestyle choices before conception to mitigate risks associated with insufficient weight gain during pregnancy.

To improve gestational weight management, it is essential to:

- Tailor dietary and lifestyle interventions to respect cultural and religious practices that may affect weight gain patterns during pregnancy.
- Encourage partner involvement in maternal health education and support, highlighting the influence of household socio-economic factors on maternal dietary behaviors.
- Implement workplace health programs that support pregnant women in physically demanding jobs, with a focus on nutrition and stress management.
- Regularly monitor hemoglobin levels and nutritional status to identify and address risks of inadequate weight gain early in pregnancy.
- Educate women on the negative impact of alcohol consumption on maternal and fetal health, emphasizing its effect on gestational weight and overall well-being.

By integrating these recommendations into prenatal care and public health initiatives, healthcare providers can better support pregnant women in achieving healthy weight management goals, thereby enhancing maternal and fetal health outcomes. This structured approach not only addresses the complexities of weight gain during pregnancy but also promotes a holistic understanding of factors influencing maternal health, contributing to improved overall pregnancy outcomes.

## Supporting information

S1 Questionnaires Raw data for PLoS ONE. (ODS)
